# Cell death mechanisms of the anti-cancer drug etoposide on human cardiomyocytes isolated from pluripotent stem cells

**DOI:** 10.1007/s00204-018-2170-7

**Published:** 2018-02-03

**Authors:** Harshal Nemade, Umesh Chaudhari, Aviseka Acharya, Jürgen Hescheler, Jan Georg Hengstler, Symeon Papadopoulos, Agapios Sachinidis

**Affiliations:** 1 Institute of Neurophysiology and Center for Molecular Medicine Cologne (CMMC), University of Cologne (UKK), Robert-Koch-Str. 39, 50931 Cologne, Germany; 2 Leibniz Research Centre for Working Environment and Human Factors, Technical University of Dortmund (IfADo), 44139 Dortmund, Germany; 3 Center of Physiology and Pathophysiology, Institute of Vegetative Physiology, University of Cologne, Robert-Koch-Str. 39, 50931 Cologne, Germany

**Keywords:** Non-animal testing, Cardiotoxicity, Safety assessment, Human pluripotent stem cells, Cardiomyocytes, Apoptosis, Calcium

## Abstract

Etoposide (ETP) and anthracyclines are applied for wide anti-cancer treatments. However, the ETP-induced cardiotoxicity remains to be a major safety issue and the underlying cardiotoxic mechanisms are not well understood. This study is aiming to unravel the cardiotoxicity profile of ETP in comparison to anthracyclines using physiologically relevant human pluripotent stem cell-derived cardiomyocytes (hPSC-CMs). Using xCELLigence real-time cell analyser (RTCA), we found that single high dose of ETP induces irreversible increase in hPSC-CMs beating rate and decrease in beating amplitude. We also identified 58 deregulated genes consisting of 33 upregulated and 25 downregulated genes in hPSC-CMs after ETP treatment. Gene ontology (GO) and pathway analysis showed that most upregulated genes are enriched in GO categories like positive regulation of apoptotic process, regulation of cell death, and mitochondria organization, whereas most downregulated genes were enriched in GO categories like cytoskeletal organization, muscle contraction, and Ca^2+^ ion homeostasis. Moreover, we also found upregulation in 5 miRNAs (has-miR-486-3p, has-miR-34c-5p, has-miR-4423-3p, has-miR-182-5p, and has-miR-139-5p) which play role in muscle contraction, arginine and proline metabolism, and hypertrophic cardiomyopathy (HCM). Immunostaining and transmission electron microscopy also confirmed the cytoskeletal and mitochondrial damage in hPSC-CMs treated with ETP, as well as noticeable alterations in intracellular calcium handling and mitochondrial membrane potential were also observed. The apoptosis inhibitor, Pifithrin-α, found to protect hPSC-CMs from ETP-induced cardiotoxicity, whereas hPSC-CMs treated with ferroptosis inhibitor, Liproxstatin-1, showed significant recovery in hPSC-CMs functional properties like beating rate and amplitude after ETP treatment. We suggest that the damage to mitochondria is a major contributing factor involved in ETP-induced cardiotoxicity and the activation of the p53-mediated ferroptosis pathway by ETP is likely the critical pathway in ETP-induced cardiotoxicity. We also conclude that the genomic biomarkers identified in this study will significantly contribute to develop and predict potential cardiotoxic effects of novel anti-cancer drugs in vitro.

**Electronic supplementary material:**

The online version of this article (10.1007/s00204-018-2170-7) contains supplementary material, which is available to authorized users.

## Introduction

Etoposide (ETP) is a podophyllotoxin derivative with a broad spectrum of antitumor activity, reported as one of the most active single agents in small-cell bronchus carcinoma and is very active in testicular carcinoma, choriocarcinoma and neuroblastoma. ETP inhibits cancer cell growth mainly via inhibition of Topoisomerase II (TopII) resulting in single and double-stranded breaks. It binds reversibly to TopoII α/β to block religation of the cleaved DNA strand, and as the binding is transient and non-covalent, enzyme activity is rapidly restored upon clearance of the compound and religation of DNA is completed (D’Arpa and Liu 1989; Soubeyrand et al. 2010; Willmore et al. 1998). It also reported to inhibit nucleoside transfer of DNA and RNA synthesis, protein synthesis, and microtubular assembly (Schmoll et al. 1981).

In clinic, ETP in combination with cyclophosphamide and vincristine (CEV) showed no cardiotoxicity and no potentiating neurotoxicity compared to Doxorubicin (DOX) in combination with cyclophosphamide and vincristine (CDV) in the treatment of small-cell lung cancer (SCLC) (Comis 1986; Hong et al. 1989). Similarly, ETP in combination with several other drugs like lomustine, methotrexate, and prednisone also proposed to be first-line therapy in patients with non-Hodgkin’s lymphoma (NHL) with no major cardiotoxicity (Dorigo et al. 1993). The previous studies have reported that ETP as a cardio-safe drug with minimum or no cardiotoxicity unlike other Topo II inhibitors like DOX, Daunorubicin (DAUNO), and Mitoxantrone (MITO). However, some reports indicate ETP-induced cardiotoxic side effects with hypotension being the most common (Cohen et al. 1977) along with development of myocardial infarction and vasospastic angina (Bernard et al. 2006; Escoto et al. 2010; Rodriguez et al. 1995; Yano and Shimada 1996). ETP has also been reported to have synergistic toxic effects on cardiac electrical activity when combined with Cisplatin (Canobbio et al. 1986; Petrella et al. 1989; Shoji et al. 2006). These inconclusive findings not only demands more efforts towards understanding the cardiotoxicity associated with ETP, but also highlights the limitations of current in vitro cardiotoxicity models.

Even though various in vitro and in vivo assays have been developed to assess the cardio safety of lead compounds, they often fail to correctly predict the actual adverse cardiac effects because of interspecies variations or lack of human-relevant model systems. Levels of diagnostic biomarkers like cTnT and cTnI in blood can be assessed easily, but they are released only after chronic cardiac tissue damage and proved to be inconclusive. For example, DOX-induced myocardial damage may appear in endomyocardial biopsy specimens, but may not produce any measurable rise in cTnT or cTnI protein levels in blood (Yeh et al. 2004). Hence, there is also an urgent need for novel and reliable in vitro as well as in vivo biomarker assay that will enable immediate and extensive assessment of cardiotoxicity.

We undertook this study aiming, to unravel the cardiotoxicity profile of ETP in comparison to that of DOX, and to further appraise the in vitro biomarkers, we recently reported for anthracycline-induced cardiotoxicity. To achieve this, we used a human-relevant model, applying that human-induced pluripotent stem cells (hiPSC) derived cardiomyocytes (hiPSC-CMs or CMs) and set of in vitro biomarkers constituting differentially regulated 84 genes and 14 miRNAs (Chaudhari et al. 2016a, b) as well as metabolites (Chaudhari et al. 2017). More recently, we could predict cardiotoxicity of cosmetic compounds using the anthracycline regulating genomic biomarkers in combination with functional readout assays (Chaudhari et al. 2018). Here, the hiPSC-CMs were exposed to high single doses of 10, 15, and 30 µM of ETP for 48 h. Cardiotoxicity in terms of changes in parameters such as cell index (CI), beating rate, and beating amplitude was monitored by xCELLigence Real-Time Cell Analyser (RTCA) cardio system. Identification of differentially regulated genes and miRNA was achieved using custom RT^2^ profiler PCR arrays and quantitative real-time PCR (qRT-PCR), respectively. Cellular toxicity was assessed with LDH leakage assay, measurements of the cellular ATP and reactive oxygen species (ROS). The effects of ETP on cell morphology and cellular cytoskeleton structure have been assessed by immunostaining and transmission electron microscopy (TEM). The effects of ETP on calcium handling and mitochondrial membrane potential have been investigated using Rhod-2AM and JC-1 fluorescence imaging.

## Materials and methods

### Chemicals

Etoposide (E1383) was obtained from Sigma-Aldrich Chemie GmbH (Germany), Liproxstatin-1 (S7699) was obtained from Selleckchem (Germany), and Pifithrin-α hydrobromide (2653) was obtained from Tocris (United Kingdom). 10 mM stock solution (in DMSO) was made and stored as small volume aliquots in tightly sealed sterile tubes at − 80 °C. Drug dilutions were performed in pre-warmed (37 °C) iCell-MM prior to each drug exposure.

### Cardiomyocyte cell culture

Purified human iCell Cardiomyocytes^®^ (Cellular Dynamics International, Madison, WI, USA), which are derived from hiPSCs, were used for all the experiments. The cardiomyocytes were supplied as a cryopreserved single cell suspension of ~ 98% pure population comprising mixture of electrically active atrial-, nodal-, and ventricular-like myocytes. These cells exhibit typical biochemical, electrophysiological, and mechanical characteristics of normal human heart cells with expected responses upon exposure to exogenous agents. For functional studies, cryopreserved hiPSC-CMs were thawed in iCell cardiomyocytes plating medium (iCell-PM, Cellular Dynamics International, Madison, WI, USA) and directly plated on a fibronectin-coated (5 µg/cm^2^, 2 h at 37 °C) E-plate Cardio 96 (ACEA Biosciences, San Diego, CA, USA) at approximately a 25 × 10^3^ cells per well density using iCell-PM. For other studies, thawed cells were plated on fibronectin-coated (5 µg/cm^2^, 2 h at 37 °C) 6-well or 96-well plates at a density of 0.4 × 10^6^ or 25 × 10^3^ cells per well, respectively. From day 2 onwards, cells were maintained in iCell cardiomyocyte Maintenance Medium (iCell-MM, Cellular Dynamics International, Madison, WI, USA), with a fresh medium change after every 2 days. The cardiomyocytes were cultured in a standard cell culture incubator at 5% CO_2_, 37 °C.

### The xCELLigence RTCA Cardio system

The xCELLigence RTCA Cardio system (ACEA Biosciences, San Diego, CA, USA) is an impedance-based platform for monitoring the real-time beating function of cardiomyocytes. The E-plate Cardio 96 (ACEA Biosciences, San Diego, CA, USA) xCELLigence plates were equilibrated using iCell-PM (50 µl per well) and inserted into the xCELLigence station to measure background impedance and to ensure that all wells and connections were working within acceptable limits. After equilibration, the cells were harvested and seeded in required density. Impedance measurements were monitored at regular time intervals. The amount of growth area covered in an E-plate Cardio 96 due to cell adhesion was represented as the Cell Index (CI). A high CI indicates more cell adhesion and vice versa. The raw data and statistical information, such as mean and SD for the parameters like CI, beating rate, amplitude, normalized CI, normalized beating rate, and normalized amplitude, were acquired using RTCA Cardio software version 1.0 (ACEA Biosciences, Inc., San Diego, CA, USA).

### RT^2^ profiler arrays for mRNA expression analysis

To analyze the mRNA expression, cell samples were homogenized with QIAzol lysis reagent (QIAGEN, Hilden, Germany), and the total RNA was extracted using the miRNeasy Mini Kit (QIAGEN, Hilden, Germany) according to the manufacturer’s instructions. Extracted RNA was assessed for purity and quantity using Nanodrop (ND-1000, Thermo Fisher, Germany). 300–500 ng of total RNA was used for cDNA synthesis using RT^2^ First Strand kit (Qiagen, Hilden, Germany) according to the manufacturer’s instructions. Quantitative comparison of mRNA levels was performed using custom made RT^2^ Profiler PCR array (96-well plate) (Qiagen, Arch Toxicol Hilden, Germany) containing 84 target genes, 5 housekeeping genes, 1 genomic DNA control, 3 reverse transcription controls, and 3 positive PCR controls (all 84 target genes are shown in Supplementary Table No. 1). The qRT-PCR was performed using RT^2^ SYBR^®^ Green ROX™ qPCR master mix (Qiagen, Hilden, Germany) in an Applied Biosystems 7500 FAST Real-Time PCR System in accordance with the manufacturer’s recommended thermal cycling conditions. The relative gene expression analysis was performed using the 2^−ΔΔ*C*t^ method with the RT^2^ PCR array data analysis online tool. Expression data were normalized using the geometric mean of five housekeeping genes, *ACTB, B2M, GAPDH, HPRT1*, and *RPLP0*. A cut-off fold change value of 1.9 was set for significantly deregulated genes and later used to generate the gene list used for Venn diagram analysis.

### Quantitative real-time PCR for microRNA expression analysis

The microRNA expression analysis was performed as we previously reported (Chaudhari et al. 2016a). In brief, 500 ng of total RNA was used for cDNA synthesis with qScript™ microRNA cDNA Synthesis Kit (Quanta Biosciences, Gaithersburg, USA) following the manufacturer’s instructions. The cDNA was diluted as per manufactures instructions and used as a template for qPCR. The amplification of miRNA was performed using the PerfeCTa^®^ microRNA assay primer, the PerfeCTa^®^ Universal PCR primer and the PerfeCTa^®^ SYBR^®^ Green SuperMix, Low ROX™ Kit (Quanta Biosciences, Gaithersburg, USA) as per the manufacturer’s instructions. Quantitative PCR was carried out on an Applied Biosystems 7500 FAST Real-Time PCR System. Relative miRNA levels were calculated using the ΔΔ*C*_t_ method, and RNU6 was used as the miRNA PCR control.

### Lactate dehydrogenase leakage assay

Cell cytotoxicity/membrane damage induced by ETP was assessed by lactate dehydrogenase (LDH) leakage into the culture medium. The iCell-CMs were seeded on a fibronectin-coated (5 µg/cm^2^, 2 h at 37 °C) 96-well plate at a cell density of 20 × 10^3^ cells per well in quadruplets. Cells then exposed to 10, 15, and 30 µM of ETP for 48 h. Following the exposure, the culture medium was aspirated and centrifuged at 3000 rpm for 5 min to obtain a cell free supernatant. The activity of LDH released into the medium was estimated using Pierce™ LDH Cytotoxicity Assay Kit (Thermo Scientific™) according to the manufacturer’s instructions. The absorbance from formazan at 490 nm was recorded using a Softmax Pro M5e 96-well plate reader (Molecular Devices, Sunnyvale, CA, USA). The raw data were analyzed and presented as percent cytotoxicity with respect to that of control with ± SD.

### Measurement of ATP in cell extracts

ATP is an important marker for cell viability and present in all metabolically active cells. Its concentration declines very rapidly when the cells undergo necrosis or apoptosis. To measure the changes in intracellular ATP after ETP treatment, cell extracts for hPSC-CMs treated with 10, 15, and 30 µM for 48 h were analyzed using ATPlite™ assay kit (PerkinElmer, Germany) according to the manufacturer’s instructions. The light intensity produced was measured at 560 nm using Softmax Pro M5e 96-well plate reader (Molecular Devices, Sunnyvale, CA, USA). Data were analyzed and represented as percent ATP as compared to that of control.

### Immunostaining

To identify whether the treatment of ETP caused any alterations in cytoskeletal assembly of CMs, we performed immunofluorescence staining of cardiac-specific proteins. Cells from ETP-treated and control groups were fixed with ice-cold 99% Methanol at − 20 °C for 10 min followed by permeabilization and fixing of cells with 0.3% Triton X-100 for 20 min and with 5% Bovine Serum Albumin for 1 h at room temperature, respectively. Cells were then incubated with anti-sarcomeric alpha actinin (α-actinin) and anti-cardiac troponin T (cTnT) antibodies at 37 °C for 1 h. All the antibodies were purchased from Abcam (Abcam, Cambridge, UK) and diluted to working concentration of 1:200. After three washes with PBS^+/+^, primary antibodies were then detected using species-matched respective Alexa Fluor-488/568-conjugated secondary antibodies (Invitrogen, Darmstadt, Germany) with 1 h incubation at 37 °C. The cells were washed three times with PBS^+/+^ for 5 min and then mounted with Prolong^®^ Gold anti-fade mount with DAPI (Invitrogen, Darmstadt, Germany). The images were acquired with an Axiovert 200 fluorescence microscope and Axiovision 4.3 software (Carl Zeiss).

### Measurement of ROS

Dihydroethidium (DHE) staining was used to assess the production of ROS in hiPSC-CMs after ETP treatment. DHE is a lipophilic, cell-permeable dye that undergoes oxidation in the presence of superoxide to form ethidium bromide, which then forms an irreversibly complex with the double-stranded DNA. The amplified red fluorescence of ethidium then can be visualized under microscope as a punctate nuclear staining indicating ROS production (Benov et al. 1998; Nijmeh et al. 2010). The ETP-treated hiPSC-CMs were incubated with DHE (5 µM) for 30 min at room temperature. Excess DHE was then washed out and cells were mounted onto a slide with Prolong^®^ Gold anti-fade mount with DAPI (Invitrogen, Darmstadt, Germany). The images were acquired with an Axiovert 200 fluorescence microscope and Axiovision 4.3 software (Carl Zeiss).

### Transmission electron microscopy

For TEM study, hiPSC-CMs were seeded onto fibronectin-coated ACLAR® embedding film (Ted Pella, Inc. USA) in 96-well plate and exposed to ETP for 48 h. Post-drug exposure cells were washed with DPBS (+/+) and fixed using 2% glutaraldehyde and 2% formaldehyde in 0.1 M cacodylate buffer (pH 7.3) for 2 h at 4 °C, post-fixed in 1% OsO4 for 30 min at 4 °C in dark. After washing, cell samples were dehydrated in graded ethanol and embedded in to Epon (Sigma) (20 g Epoxy, 11 g DDSA, 9 g NMA, 0.8 g DMP-30) for 48–72 h at 62 °C. Ultrathin (~ 70 nm) sections were obtained using Leica EM UC6 Ultramicrotome (Leica, Germany) and double-stained with uranyl acetate followed by lead citrate. Ultrastructural analysis was done using a transmission electron microscope (EM109, Zeiss, Germany) equipped with frame transfer CCD camera.

### Calcium imaging

The hiPSC-CMs were seeded onto fibronectin-coated thin glass bottom (0.085–0.115 mm) 35 mm dish (In Vitro Scientific) and treated with ETP for 48 h. Then, the cells were washed and incubated with 2 µM Rhod-2, AM Ca^2+^ dye (Invitrogen) for 30 min at 37 °C, and washed. Rhod-2-labeled cells were then observed and fluorescence imaging was performed at room temperature, with an inverted confocal laser-scanning microscope (FV1000, Olympus) operating in the line-scan mode, equipped with a 60× oil immersion objective. Upon recording a line scans, background subtraction was applied for all data sets, by defining the average (auto-) fluorescence intensity of an extracellular area as the background. The data were then subjected to a fitting procedure which adjusted the parameters of a Weibull function implemented in Sigma Plot (Version 8.04, SPSS). A self-made macro in Excel (Microsoft) calculated the Ca^2+^ transient amplitude (*F*/*F*_0_, where *F*_0_ is the averaged background-corrected resting fluorescence intensity), time-to-peak (TTP), the maximum steepness ([Δ*F*/Δ*T*] max), full-width at half-maximum (FWHM) and T90%. Data were represented as ± SEM of 25 measurement (*n* = 25) for each group.

### Mitochondrial membrane potential (*m*∆*ψ*) assay

We used JC-1-staining method (Nuydens et al. 1999) to investigate the loss of mitochondrial membrane potential in response to ETP treatment (10, 15, and 30 µM) for 48 h, DOX (1 µM) was used as a positive control. In brief, hiPSC-CMs were seeded onto fibronectin-coated thin glass bottom (0.085–0.115 mm) 35 mm dish (In Vitro Scientific) and treated with ETP for 48 h. Then, the cells were washed and incubated with 10 µM JC-1 dye (AAT Bioquest, CA) diluted in fresh medium for 30 min in 37 °C, 5% CO_2_ incubator, and washed. JC-1 emissions after excitation at the 488 nm were captured at 525 nm (JC-1 monomers; green) and 590 nm (JC-1 aggregates; orange). The images were captured using inverted confocal laser-scanning microscope (FV1000, Olympus).

### Statistical analysis

Experimental results are expressed as mean ± SEM. Two-tailed Student’s *t* test was used to calculate statistical significance and p values ≤ 0.05 were considered as statistically significant.

## Results

### Single high dose of etoposide leads to arrhythmic beating and cytotoxicity in hiPSC-CMs

**Fig. 1:**
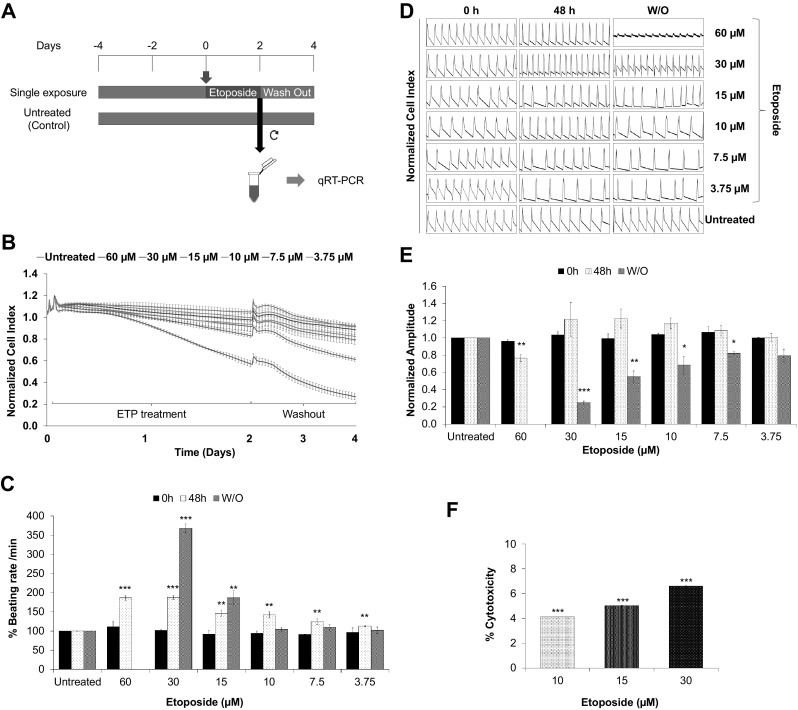
Single high dose of etoposide induces arrhythmic beating and cytotoxicity in hiPSC-CMs. **a** Schematic representation and experimental setup of the in vitro cardiotoxicity test model. For functional studies, the synchronously beating hiPSC-CMs in the E-plate Cardio 96 were exposed to ETP (single high-dose exposure) for 48 h. After exposure, the ETP was washed out and the cells were further incubated for 48 h. The effects of ETP on hPSC-CMs functional characteristics were monitored by the xCELLigence RTCA Cardio system. For qRT-PCR studies, RNA from ETP-treated and untreated control cells were harvested at day 2. **b**–**e** Functional studies of ETP-treated hiPSC-CMs. The representative graphs display, **b** normalized CI values showing ETP-induced cytotoxicity (*n* = 3, error bars represent ± SEM), **c** % beating rate alterations induced by single dose of ETP in hiPSC-CMs (*n* = 3, error bars represent ± SEM) (*t* test, *p < 0.05, **p < 0.01, ***p < 0.001), **d** representative 12 s beating traces of hiPSC-CMs before, during and after the ETP treatment,** e** normalized amplitude showing significant drop after ETP treatment (*n* = 3, error bars = ± SEM) (*t* test, *p < 0.05, **p < 0.01, ***p < 0.001). **f** ETP-induced cytotoxicity was assessed by LDH leakage assay. The graph shows % cytotoxicity induced by ETP compared to untreated control (*n* = 3, error bars represent ± SEM) (*t* test, *p ≤ 0.05, **p ≤ 0.01, ***p ≤ 0.001)

The hiPSC-CMs were thawed and seeded onto fibronectin-coated E-Cardio plate in iCell-PM and maintained in iCell-MM. Then, synchronously beating hPSC-CMs treated with ETP according to the timeline, as represented in Fig. 1a. The hPSC-CMs were treated with ETP from day 0 to day 2 followed by 48 h drug wash out. Raw data of CI and beating profile were obtained from the xCELLigence RTCA Cardio system for the assessment of the alterations in hPSC-CMs functional properties. In addition, to perform qRT-PCR analysis of genomic and miRNA biomarkers, ETP-treated hPSC-CMs were harvested on day 2 with respective untreated controls. Our initial data show that single dose of > 30 µM ETP had very high cytotoxicity towards hPSC-CMs and leads to drastic drop in the CI (Fig. 1b), whereas single dose of < 10 µM ETP had no significant effect on CI or hPSC-CMs’ functional properties. Interestingly, 30 and 15 µM ETP caused irreversible increase in the beating rate of hPSC-CMs leading to alterations in the beating profile and arrhythmic beating (Fig. 1c, d). Furthermore, 10 µM ETP also showed initial increase in beating rate and changes in beating profile, but hPSC-CMs were able to recover to basal levels after drug wash out. Similarly, we also observed alterations in the beating amplitude of hPSC-CMs due to ETP treatment (Fig. 1e). The level of extracellular lactate dehydrogenase (LDH) was used as an indicator of membrane damage and cell death (Chan et al. 2013). We observed significant and dose-dependent increase in extracellular LDH level post-ETP treatment, indicating membrane damage and stimulation of apoptotic cell death in hiPSC-CMs (Fig. 1f). Based on these findings, ETP at 10, 15, and 30 µM was considered the most effective concentration and used for further cardiotoxicity assessments. The raw data from quadruplet experiment were acquired, analyzed, and represented as percent cytotoxicity with ± SEM (Chan et al. 2013).

### The qRT-PCR based analysis of hiPSC-CMs treated with single high dose of etoposide identifies cluster of deregulated genomic and miRNA markers

**Fig. 2:**
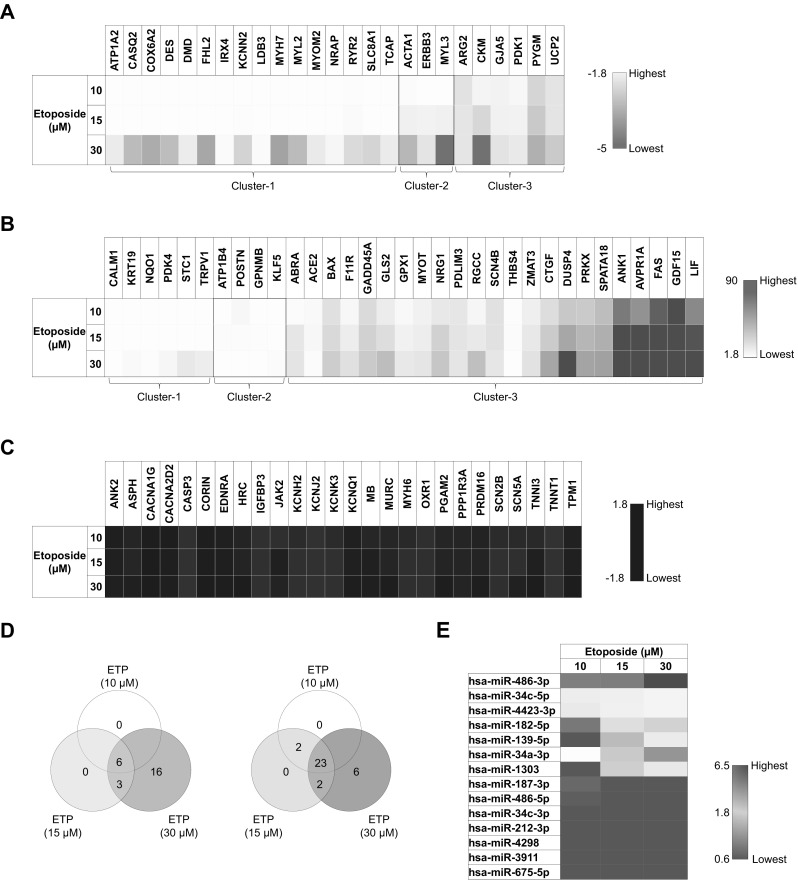
Etoposide deregulates cluster of genomic and miRNA biomarkers in hiPSC-CMs. Heat maps showing ETP-induced deregulation of genomic and miRNA markers in CMs. **a** Represents genes downregulated after ETP treatment (*n* = 3, fold change ≤ − 1.8). **b** Represents genes upregulated after ETP treatment (*n* = 3, fold change ≥ 1.8). **c** Represents genes with no significant change in expression level after ETP treatment (*n* = 3). **d** Venn diagram showing commonly deregulated genes in groups with different ETP concentrations (*n* = 3). **e** Represents deregulated miRNA markers (*n* = 3)

Applying wide genome microarrays, we recently reported array of 84 genes (Chaudhari et al. 2016b) and 14 miRNAs (Chaudhari et al. 2016a) as potential biomarkers for the assessment of drug-induced cardiotoxicity using hiPSC-CMs. These biomarkers include 50 down- and 34 upregulated genes and 4 down- and 10 upregulated miRNAs. To explore the underlying mechanisms involved in ETP-induced cardiotoxicity, we performed qRT-PCR analysis of these biomarkers from hPSC-CMs treated with single high dose of (10, 15, and 30 µM) ETP for 48 h. Genes with at least 1.8-fold changes were identified and grouped based on fold change values. Three groups of genes were identified; group-1 represents downregulated genes (Fig. 2a); group-2 represents upregulated genes (Fig. 2b); and group-3 represents genes with no change in fold expression values (Fig. 2c). Genes from group-1 and -2 were further sub-categorized in three clusters based on concentration of drug and fold change values (Fig. 2d). Altogether, out of 84 genes, 58 were deregulated consisting 25 down- and 33 upregulated genes. Fold change values of all the deregulated genes are shown in Supplementary table (1). To further investigate the biological functions and molecular pathway involvement of these genes, Database for Annotation, Visualization and Integrated Discovery (DAVID) was used for functional annotation and gene ontology (GO) clustering (Dennis et al. 2003). KEGG pathway and GO analysis showed that downregulated genes from group-1 were enriched in 4 KEGG pathways and GOs including muscle contraction, cardiac muscle contraction, calcium ion transport from endoplasmic reticulum to cytosol, and cytoskeleton organization (Table 1). Similarly, upregulated genes from group 2 were mainly enriched in 1 KEGG pathway and GOs like regulation of cell death, regulation of apoptotic process, programmed cell death, positive regulation of apoptotic process, and mitochondrion organization (Table 2). In addition, we also identified 5 out of 14 miRNAs; hsa-miR-486-3p, hsa-miR-34c-5p, hsa-miR-4423-3p, hsa-miR-182-5p, and hsa-miR-139-5p deregulated after ETP treatment (Fig. 2e). Fold change values of all the deregulated miRNAs are shown in Supplementary table (2). The hsa-miR-486-3p, hsa-miR-182-5p, and hsa-miR-139-5p showed dose-dependent upregulation in fold expression value, whereas hsa-miR-34c-5p and hsa-miR-4423-3p remained twofold upregulated with no significant deviation in fold expression at all three test concentration. Target genes of each upregulated miRNA were then identified using the miRWalk 2.0 bioinformatics tool (Dweep et al. 2011) and matched with ETP-induced downregulated genes as we previously reported (Chaudhari et al. 2016a). Common genes were analyzed for the enrichment of GO and KEGG pathways using the DAVID. As shown in Supplementary data 1, the downregulated genes were mainly enriched in GO terms such as the muscle contraction, regulation of heart contraction, actin filament-based process, and cardiac muscle cell action potential and KEGG pathways such as cardiac muscle contraction, arginine and proline metabolism, and hypertrophic cardiomyopathy (HCM). Taken together, these findings indicate that ETP induces cardiotoxicity mainly via cytoskeletal damage and activation of cell death via apoptosis and death receptor signaling in CMs. In addition, we also show that using combination of hiPSC-CMs and cardiotoxicity biomarkers, we can assess the cardiotoxicity of known or novel chemical entities.

**Table 1:** Significantly enriched GO categories and pathways by group-1 genes (downregulated genes after etoposide treatment)

Components	Gene count	*p* value	Representative genes
GO terms: Group-1			
GO:0006936—muscle contraction	15	7.00E−19	SLC8A1, MYL2, ACTA1, TCAP, MYL3, MYH7, ATP1A2, GJA5, DES, MYOM2, ARG2, DMD, KCNN2, RYR2, CASQ2
GO:0060048—cardiac muscle contraction	11	2.49E−16	SLC8A1, MYL2, TCAP, MYL3, DMD, KCNN2, RYR2, MYH7, ATP1A2, GJA5, CASQ2
GO:0060047—heart contraction	11	3.52E−13	SLC8A1, MYL2, TCAP, MYL3, DMD, KCNN2, RYR2, MYH7, ATP1A2, GJA5, CASQ2
GO:0086065—cell communication involved in cardiac conduction	6	4.47E−09	SLC8A1, KCNN2, RYR2, ATP1A2, GJA5, CASQ2
GO:0010882—regulation of cardiac muscle contraction by calcium ion signaling	5	2.33E−08	SLC8A1, DMD, RYR2, ATP1A2, CASQ2
GO:1903514—calcium ion transport from endoplasmic reticulum to cytosol	5	3.32E−08	SLC8A1, DMD, RYR2, ATP1A2, CASQ2
GO:0042391—regulation of membrane potential	8	3.58E−07	SLC8A1, UCP2, DMD, KCNN2, RYR2, ATP1A2, GJA5, CASQ2
GO:0007010—cytoskeleton organization	7	0.0036	DES, MYL2, TCAP, ACTA1, DMD, LDB3, CASQ2
GO:0015992—proton transport	3	0.0184	UCP2, COX6A2, ATP1A2
KEGG pathways: Group-1			
hsa04260:Cardiac muscle contraction	6	3.63E−07	MYL2, MYL3, COX6A2, RYR2, MYH7, ATP1A2
hsa05410:Hypertrophic cardiomyopathy (HCM)	5	1.87E−05	DES, MYL2, MYL3, DMD, RYR2
hsa05414:Dilated cardiomyopathy	5	2.51E−05	DES, MYL2, MYL3, DMD, RYR2
hsa05412:Arrhythmogenic right ventricular cardiomyopathy (ARVC)	3	0.010025	DES, DMD, RYR2

**Table 2:** Significantly enriched GO categories and pathways by group-2 genes (upregulated genes after etoposide treatment)

Components	Gene count	*p* value	Representative genes
GO terms: Group-2			
GO:0010941—regulation of cell death	14	1.61E−06	TRPV1, ZMAT3, PDK4, GLS2, GPX1, CTGF, BAX, RGCC, FAS, NQO1, GPNMB, GDF15, NRG1, GADD45A
GO:0042981—regulation of apoptotic process	13	5.02E−06	GLS2, GPX1, TRPV1, CTGF, ZMAT3, BAX, RGCC, PDK4, FAS, GDF15, NRG1, NQO1, GADD45A
GO:0033554—cellular response to stress	14	9.76E−06	GPX1, TRPV1, CTGF, ZMAT3, BAX, SPATA18, RGCC, PDK4, AVPR1A, STC1, FAS, NQO1, GADD45A, THBS4
GO:0043065—positive regulation of apoptotic process	8	8.22E−05	TRPV1, CTGF, ZMAT3, BAX, RGCC, FAS, NQO1, GADD45A
GO:0012501—programmed cell death	13	8.51E−05	GLS2, GPX1, TRPV1, CTGF, ZMAT3, BAX, RGCC, PDK4, FAS, GDF15, NRG1, NQO1, GADD45A
GO:0043408—regulation of MAPK cascade	8	2.36E−04	LIF, DUSP4, CTGF, FAS, GPNMB, GDF15, NRG1, GADD45A
GO:2001239—regulation of extrinsic apoptotic signaling pathway in absence of ligand	3	0.0042	BAX, FAS, NRG1
GO:0097191—extrinsic apoptotic signaling pathway	4	0.0088	GPX1, BAX, FAS, NRG1
GO:0010821—regulation of mitochondrion organization	4	0.0093	GLS2, GPX1, BAX, NRG1
GO:0008625—extrinsic apoptotic signaling pathway via death domain receptors	3	0.0099	GPX1, BAX, FAS
GO:0042770—signal transduction in response to DNA damage	3	0.0215	BAX, RGCC, GADD45A
GO:0007005—mitochondrion organization	5	0.0344	GLS2, GPX1, BAX, SPATA18, NRG1
KEGG pathways: Group-2			
hsa04115:p53 signaling pathway	4	7.56E−04	ZMAT3, BAX, FAS, GADD45A

### Etoposide induces cytoskeletal damage and sarcomeric deterioration in hiPSC-CMs

**Fig. 3:**
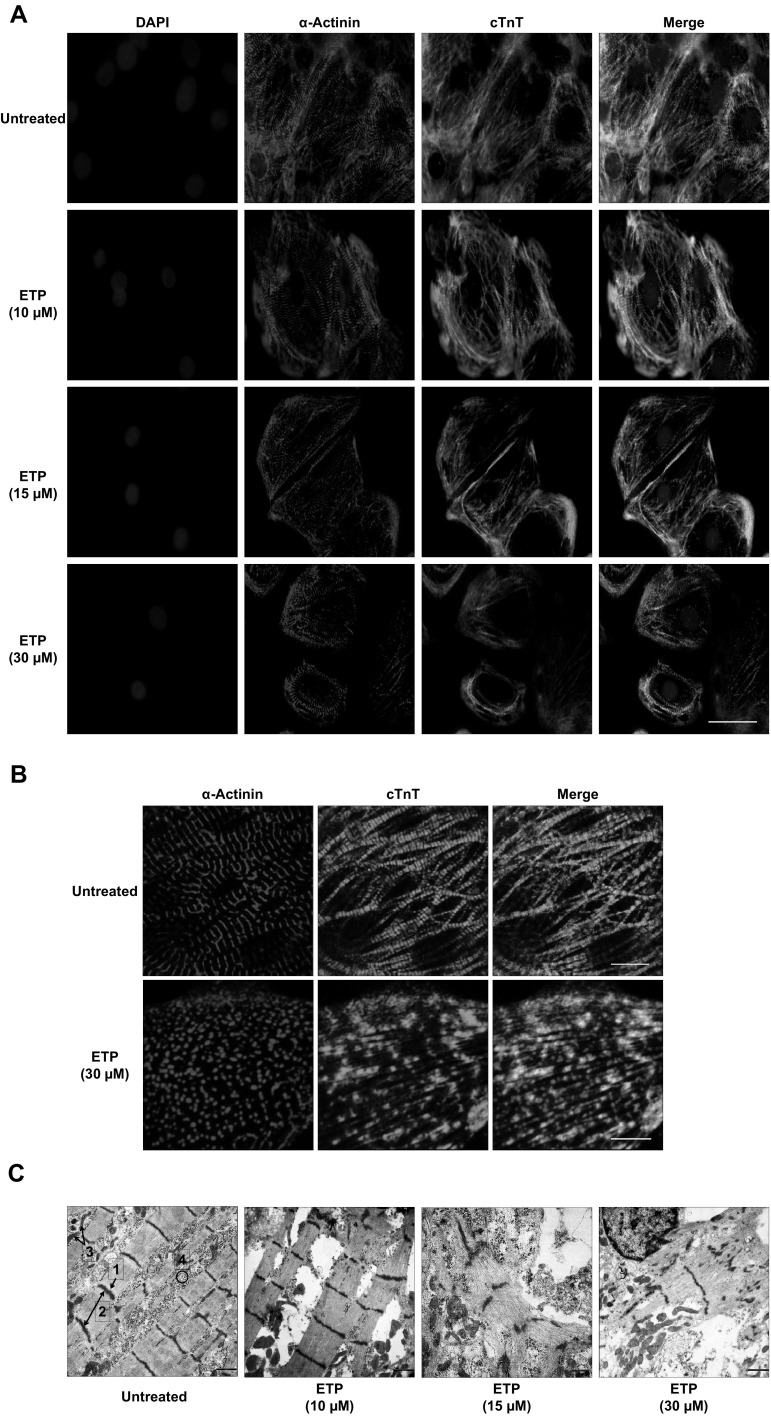
Etoposide induces cytoskeletal disorganization in hiPSC-CMs. **a, b** Immunostaining of cardiac sarcomeric α-actinin (α-Actinin) and cardiac troponin T (cTnT) in untreated and ETP-treated hiPSC-CMs. Nuclei are stained with DAPI. Scale bar represents 50, 5 µm. **c** Represents TEM images of untreated and ETP-treated hiPSC-CMs. Scale bar represents 5000 nm. (1, Z-line; 2, sarcomere; 3, mitochondria; 4, myofibril bundles)

The earliest morphological changes in iCell-CMs exposed to high concentrations of ETP were evident. Visible alterations in cell morphology, vacuolization within the sarcoplasm and alterations in cell size were observed after treatment (Fig. S1A). Even though the qRT-PCR data did not show any significant deregulation of cardiac cytoskeletal genes like MYH6, TNNI3, and TNNT1, downregulation in expression of MLY2, MYL3, MYH7, MYOM2, DES, and ACTA1 genes was observed after ETP treatment. In addition, ERBB3 and FHL2, which are important for normal cardiac development and play a crucial role in recovering cardiomyocytes from various types of cardiac insults (Erickson et al. 1997; Tran et al. 2016), were also found to be downregulated after ETP treatment. Many of these genes belong to group-1/cluster-1 and group-1/cluster-2 indicating dose-dependent cytotoxicity of ETP. Using antibodies for anti-sarcomeric alpha actinin (α-Actinin) and cardiac troponin T (cTnT), we further investigated the effect of ETP exposure on cardiac sarcomere structures. Immunostaining revealed disorganizations in cardiac sarcomere structures in hPSC-CMs treated with ETP compared to that of untreated cells (Fig. 3a). Particularly, severe cytoskeletal deterioration was observed at 30 µM ETP (Fig. 3b). Subsequent transmission electron microscopy analysis also confirmed the membrane and cytoskeletal damage including irregularities in cardiac myofibrillar arrangements in ETP-treated hPSC-CMs (Fig. 3c). Collectively, these results show that ETP exposure induces cell membrane damage and disorganization of cardiac sarcomere structures.

### Etoposide causes alterations in calcium handling in hiPSC-CMs

**Fig. 4:**
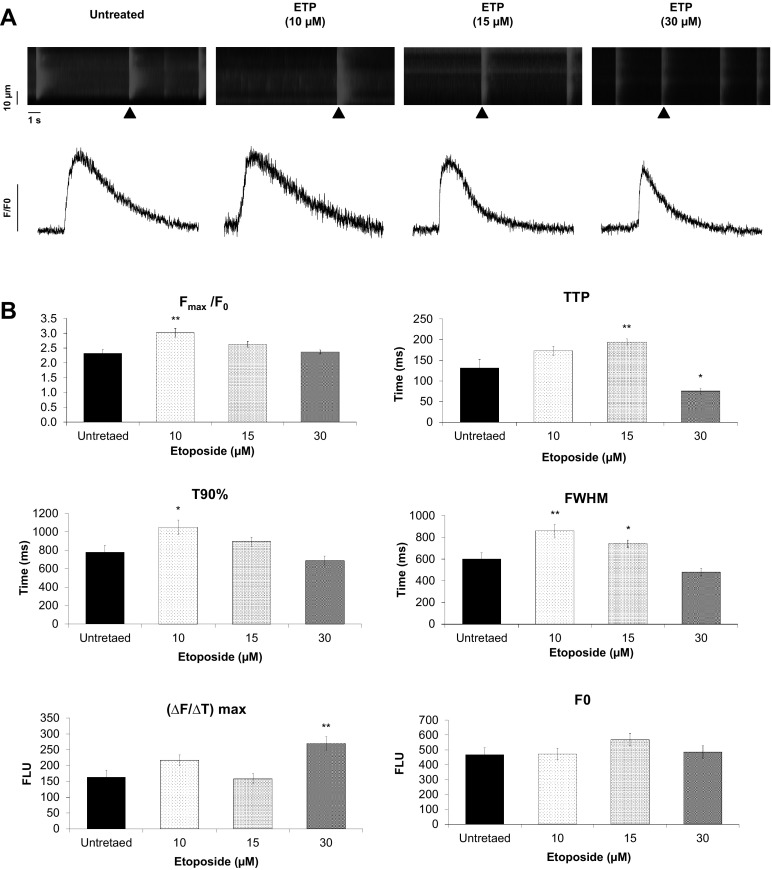
Etoposide causes alterations in calcium handling in hiPSC-CMs. **a** Confocal line-scan images showing changes in intracellular [Ca^2+^]_*i*_ in a Rhod-2, AM loaded hiPSC-CM. The images show alterations in spontaneous whole-cell Ca^2+^ transients in response to ETP treatment (upper panel). Scale bar represents, time − 1 s and distance − 10 µm. Representative tracings of spontaneous Ca^2+^ transients (black arrow head) in hiPSC-CMs from untreated and ETP-treated groups (lower panel). **b** Graphs representing Ca^2+^ transient parameters measured from hiPSC-CMs treated with ETP. *F*/*F*_0_, Ca^2+^ transient amplitude where *F*_0_ is the averaged background-corrected resting fluorescence intensity; TTP, time-to-peak; T90%, 90% recovery of *F*_max_; [Δ*F*/Δ*T*]_max_, the maximum steepness; FWHM, full-width at half-maximum. (*n* = 25, error bars represent ± SEM) (*t* test, *p ≤ 0.05, **p ≤ 0.01)

Calcium (Ca^2+^) is the critical intermediary in the process of cardiac EC-coupling, connecting hPSC-CMs depolarization with mechanical contraction. In addition to opening cardiac type ryanodine receptors (RyR2) via the mechanism of Ca^2+^-induced Ca^2+^ release (CICR), Ca^2+^ is also critical in the regulation of several other important aspects of hPSC-CMs biological processes such gene transcription. We found several genes, among them CALM1, SLC8A1, DMD, RYR2, ATP1A2, CASQ2, and ERBB3, which play important roles in cardiac Ca^2+^ handling, to be deregulated in hPSC-CMs after ETP treatment. To investigate the effect of ETP on cardiac Ca^2+^ handling in greater detail, we recorded and analyzed spontaneous Ca^2+^ transients in hPSC-CMs loaded with the Ca^2+^ indicator, Rhod-2 AM (Fig. 4a). ETP had concentration dependent, partially contrary effects on the Ca^2+^ transient amplitude and on the kinetics. The hiPSC-CMs treated with ETP at 10 and 15 µM showed a higher Ca^2+^ transient amplitude (Fig. 4b, *F*_max_/*F*_0_), a slowing in reaching the [Ca^2+^] peak (Fig. 4b, TTP) as well a prolonged presence of Ca^2+^ in the sarcoplasm (Fig. 4b, T90% and FWHM). In contrast, 30 µM ETP caused an acceleration of the ascending phase of the transient (Fig. 4b, TTP and Δ*F*/Δ*T*_max_). The kinetics of Ca^2+^ cycling in hPSC-CMs are determined in large part by the complex interplay between the type 2 ryanodine receptor (RyR2), the cardiac dihydropyridine receptor (DHPR), and the SERCA2a Ca^2+^ pump. Subtle alterations in these components through mutation, dysregulation or drug toxicity can have profound effects on the function of CMs. Our data show that ETP exposure could indeed cause alterations in the function of one or more of these critical Ca^2+^ cycling components, which may lead to the observed altered Ca^2+^ transients and which ultimately will affect the normal functionality of CMs.

### Etoposide upregulates apoptosis signaling in hiPSC-CMs

**Fig. 5:**
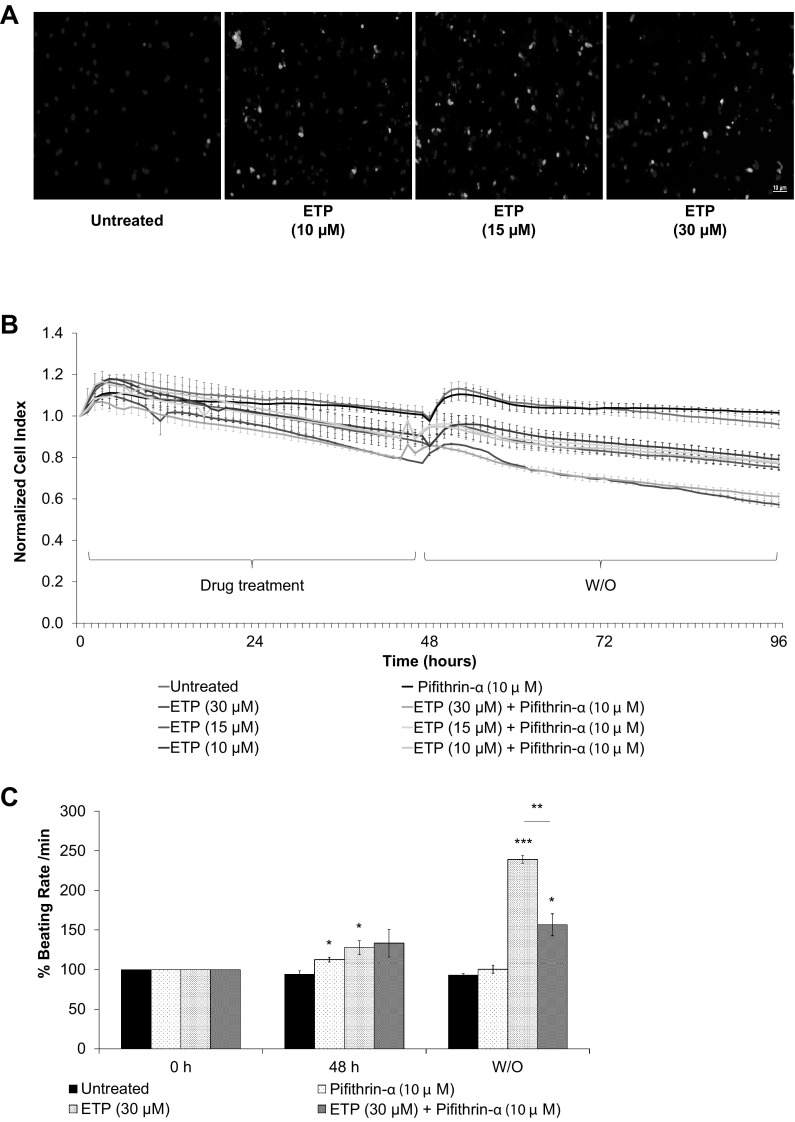
Etoposide upregulates apoptosis signaling in hiPSC-CMs. **a** Fluorescent images showing live cells (blue), apoptotic cells (green), and necrotic cells (red) in hiPSC-CMs after ETP treatment for 48 h. Scale bar represents 10 µm. **b, c** hiPSC-CMs were co-treated with ETP and 10 µM Pifithrin-α for 48 h. Real-time data of hPSC-CMs cell index and beating rate were obtained using xCELLigence RTCA system. Representative graphs display normalized CI and % beating rate values, respectively, showing Pifithrin-α had significant effect in preventing ETP-induced cardiotoxicity (*n* = 3, error bars represent ± SEM) (*t* test, *p ≤ 0.05, ***p ≤ 0.001) (see also Fig. S2A). (Color figure online)

Like many other Topoisomerase II inhibitors, ETP has been reported to induce apoptosis in thymocytes, BAF3 cell line and other cancer lines; this induction of apoptosis mainly has been linked to DNA fragmentation originated by inhibition of Topoisomerase II (Onishi et al. 1993; Walker et al. 1991). In addition, in recent study upregulation FAS has been reported as a critical mechanism involved in DOX-induced cardiotoxicity (Zhao and Zhang 2017). In accordance with these findings, our qRT-PCR data show that ETP treatment leads to upregulation of genes involved in apoptosis and death receptor signaling like BAX, FAS, RGCC, GLS2, GPX1, ZMAT3, and GADD45A, although we did not observed any significant upregulation in caspase 3 (CASP3) mRNA levels after ETP treatment. To further confirm the activation of apoptosis, we stained ETP-treated hPSC-CMs with Phosphatidylserine (PS) sensor, apopxin and 7-aminoactinomycin D (7-AAD), for late stage apoptosis and necrosis. Confocal images clearly show activation of apoptosis after 48 h of ETP treatment. Notable, increase in number of red stained nuclei was observed in hPSC-CMs treated with 30 µM ETP indicating severe membrane damage and late stage apoptosis and necrosis (Fig. 5a). To ascertain the role of apoptosis signaling in ETP-induced cardiotoxicity, we then performed RTCA experiments, where hPSC-CMs were treated with ETP in the presence of Pifithrin-α (an inhibitor of p53 transcriptional activity) and real-time data of hPSC-CMs CI and beating rate were obtained from the xCELLigence RTCA Cardio system. Pifithrin-α has been shown to protect hPSC-CMs against DOX-induced apoptosis in mouse heart (Liu et al. 2004). It inhibits apoptosis and attenuates DOX-induced elevation in Bax and MDM2 protein levels. Our RTCA data show that Pifithrin-α did not prevent ETP-induced cytotoxicity (Fig. 5b) but interestingly it did prevent the ETP-induced increase in beating rate in hiPSC-CMs (Fig. 5c). Pifithrin-α alone causes slight increase in beating rate (Fig. S2A). Altogether, these data suggest that apoptosis signaling might be the crucial player in ETP-induced cardiotoxicity and Pifithrin-α contributed to rescue the hPSC-CMs from ETP-induced cardiotoxicity.

### Etoposide induces mitochondrial damage, increase of ROS production and loss of mitochondrial membrane potential in hiPSC-CMs

**Fig. 6:**
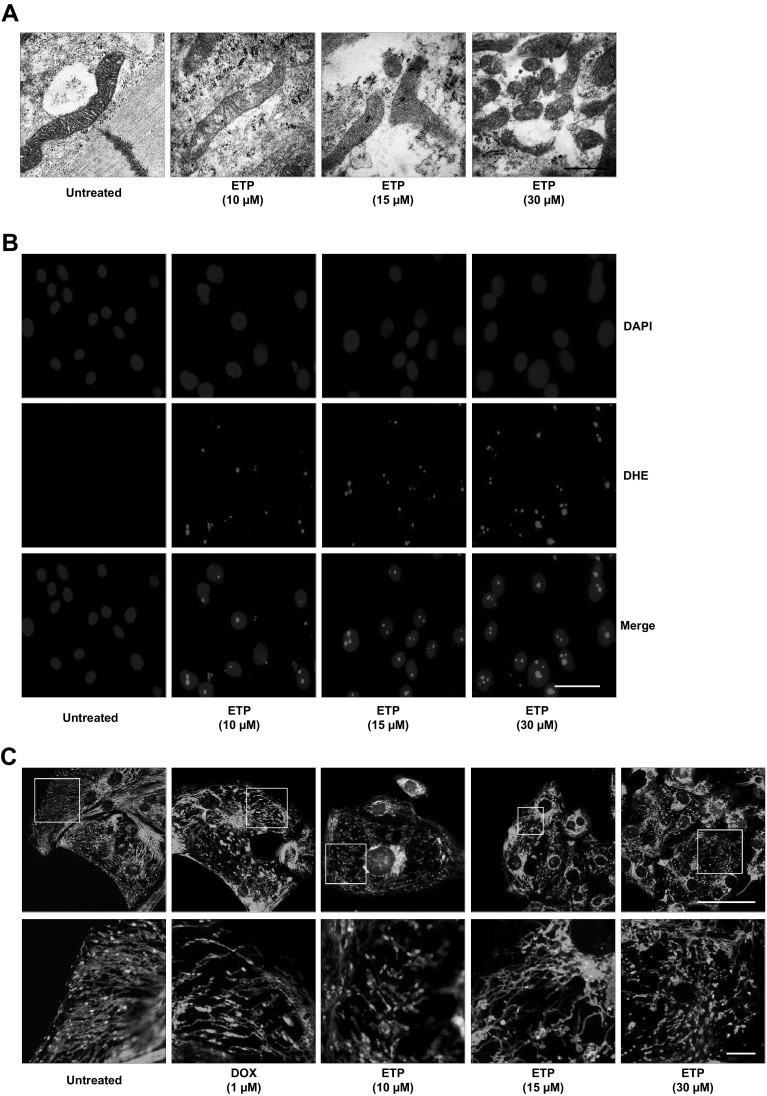
Etoposide induces mitochondrial damage, increased ROS production and loss of mitochondrial membrane potential (*m*∆*ψ*) in hiPSC-CMs. **a** TEM images showing morphological alterations in mitochondrial membrane and cristae structures in hiPSC-CMs treated with ETP compared to untreated CMs. Scale bar represents 500 nm. **b** Fluorescent images showing increase DHE staining in hiPSC-CMs treated with ETP compared to untreated CMs, indicating net increase in ROS production. Nuclei are stained with DAPI which also show increased nuclear size after ETP treatment. Scale bar represents 20 µm. **c** Determination of mitochondrial membrane potential through JC-1 staining. Mitochondria of hiPSC-CMs after incubation with JC-1 dye, illustrating the heterogeneity in mitochondrial membrane potential in the same cell. The mitochondria membrane potential was found to be interrupted after DOX (positive control) and ETP treatment, as evidenced by reduction in the JC-1 red fluorescence signal. In addition hPSC-CMs treated with 30 µm ETP for 48 h showed increased mitochondrial fragmentation further supporting the TEM data. Scale bar represents 20 µm (upper panel), 5 µm (lower panel)

Measurements of intracellular ATP levels from ETP-treated hiPSC-CMs showed drop in the ATP levels indicating inhibition of ATP production and altered mitochondrial bioenergetics function (Fig. S1B). To further assess the mitochondrial quality and structure we performed transmission electron microscopy and JC-1 staining of ETP-treated CMs. TEM imaging showed intact, elongated mitochondria with clear cristae in untreated hPSC-CMs, whereas damage to mitochondrial membrane and mitochondrial fragmentation was observed in ETP-treated CMs. As reported, ETP at 30 µM showed severe damage and fragmentation of mitochondria compared to that of an untreated cells (Fig. 6a). This mitochondrial damage can cause an imbalance between reactive oxygen species (ROS) production and removal, resulting in net increase in ROS production. To evaluate ROS production after ETP treatment we used Dihydroethidium (DHE) staining. Confocal imaging revealed that ETP-treated hPSC-CMs had dose-dependent increase in nuclear DHE staining suggesting net increase in ROS production in a dose-dependent manner (Fig. 6b). The abnormal enlargements in cell and nuclear size were previously correlated with anti-cancer drugs induced side effects (Kang et al. 2011). Nuclear staining with DAPI showed increased nuclear size in hiPSC-CMs treated with ETP indicating DNA damage and chromosomal abnormalities which could result in genomic instability. This DNA damage was also confirmed by upregulation in DNA damage response genes like RGCC, ZMAT3 and GADD45A in ETP-treated CMs. It has been shown that SPATA18 interacts with BH3 domain of NIX in ROS dependent manner and induces vacuole-like structures within mitochondria which engulf and degrade the unhealthy mitochondria (Kitamura et al. 2011). Our qRT-PCR data showed significant upregulation in SPATA18 and key genes responsible for regulation of ROS generation like NQO1, GLS2, and GPX1 and, therefore, indicate increased ROS production and mitochondrial damage in ETP-treated CMs. We next investigated the effect of ETP on mitochondrial membrane potential (*m*∆*ψ*) using JC-1 staining. Healthy mitochondria give out red fluorescence because of J aggregates of JC-1 dye and dead/apoptotic cells show green fluorescence due to lack of mitochondrial membrane potential (Mathur et al. 2000). Confocal imaging shows that like DOX (positive control) ETP exposure leads to significant loss of mitochondrial membrane potential in a dose-dependent manner compared to that of an untreated cells (Fig. 6c). Altogether, these findings indicate that increased mitochondrial damage and activation of p53-SPATA18 pathway to repair or eliminate damaged mitochondria in hiPSC-CMs after ETP treatment.

### Ferroptosis inhibitor, Liproxstatin-1 helps to improve cardiomyocytes functional properties after etoposide treatment

**Fig. 7:**
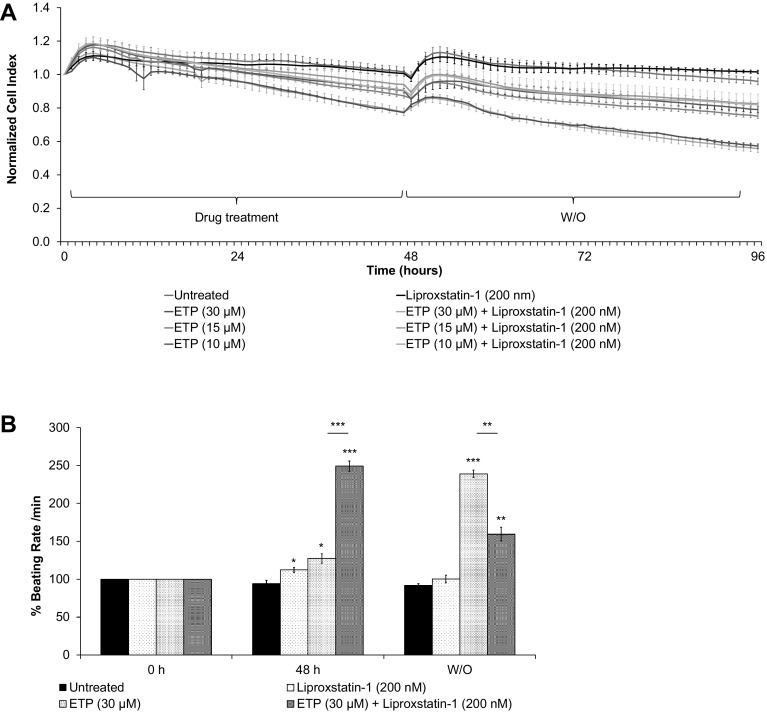
Liproxstatin-1 improves cardiomyocytes functional properties after etoposide treatment. The hiPSC-CMs were co-treated with ETP and 200 nM Liproxstatin-1 (ferroptosis inhibitor) for 48 h. Real-time data of hPSC-CMs cell index, beating rate, and beating amplitude were obtained using xCELLigence RTCA system. Representative graphs display **a** normalized CI and **b** % beating rate values, respectively, showing even though Liproxstatin-1 had no significant effect in preventing ETP-induced cytotoxicity; it significantly improved hPSC-CMs ability to recover from ETP-induced alterations in beating rate and beating amplitude (*n* = 3, error bars represent ± SEM) (*t* test, *p ≤ 0.05, **p ≤ 0.01, ***p ≤ 0.001) (see also Fig. S3A)

Ferroptosis is a form of an iron-dependent non-apoptotic cell death (Dixon et al. 2012). It is characterized by increased ROS production and morphologically by mitochondrial deformities. Smaller than normal mitochondria with reduced/vanishing mitochondrial cristae indicated likely activation of ferroptosis in ETP-treated hiPSC-CMs, this is further supported by the increased ROS production (Xie et al. 2016), p53 upregulation and GLS2 upregulation (Gao et al. 2015). To evaluate whether ferroptosis plays a role in ETP-induced cardiotoxicity, we simultaneously treated hPSC-CMs with ETP and liproxstatin-1 (a ferroptosis inhibitor). The RTCA analysis showed that the treatment of liproxstatin-1 did not show significant improvement in hPSC-CMs viability during and after ETP treatment (Fig. 7a). However, during wash out, it significantly promoted hPSC-CMs recovery from increase in beating rate induced by ETP (Fig. 7b). In addition, at lower ETP concentrations, liproxstatin-1 treated hPSC-CMs were able to fully recover from ETP-induced alterations as seen by stabilized beating rate (Fig. S2B). These data suggest that ferroptosis might be involved in ETP-induced cardiotoxicity.

## Discussion

Etoposide is a DNA topoisomerase II (TOP II) inhibitor that has significant activity against wide spectrum human cancers such as, small-cell lung cancer, non-Hodgkin’s lymphoma, stomach cancer and ovarian cancer (Hansen et al. 1977; Jiang et al. 2016; Klumpp et al. 1993). ETP inhibit TOP II-α and TOP II-β isoforms, thereby leading to genotoxicity and cell death. It has been in clinical use for more than two decades; however, clinical application of ETP are limited due to its side effects on organs like kidney and heart. Although ETP-induced arrhythmia are very rare (Kridis et al. 2013), however, it has been reported to induce atrial arrhythmia and infarction especially when combined with cytotoxic drug like cisplatin (Kucharz et al. 2016; Petrella et al. 1989). In addition, several case studies also reported myocardial infarction and vasospastic angina in ETP-treated patients (Schwarzer et al. 1991; Yano and Shimada 1996). Although the underlying mechanisms for ETP-induced cardiotoxicity and alterations in cardiac function remains to be fully explored, the previous studies on DOX-induced cardiotoxicity points-to the inhibition of TOP II-β by ETP as one of the major contributing factor.

We found that single high dose of ETP induces cytotoxicity leading to cell death and arrhythmia indicated by irreversible increase in hPSC-CMs beating rate. Current methods for evaluation of cardiotoxicity rely on studies performed using cell line overexpressing cardiac ion channels, especially the human ether-a-go–go (hERG) channel or animal models, especially mice models. Yet, cardiovascular toxicity remains a major contributing factor for post-approval withdrawal of medicines (Ferri et al. 2013). Limitations with current cardiotoxicity models demand urgent need to develop human-relevant in vitro and in vivo cardiotoxicity models. In the present study, we have established an in vitro cardiotoxicity methodology for the monitoring of early as well as chronic cardiotoxicity events associated with ETP at the cellular, functional and genomic level. In accordance with the previous studies, we found increased expression levels of ACE2, DUSP4, LIF and NRG1 in hiPSC-CMs treated with ETP making them suitable candidate for in vitro cardiotoxicity markers (Choi et al. 2012; Goulter et al. 2004; Jougasaki et al. 2003; Yan and Morgan 2011). GDF15, a stress-responsive transforming growth factor-beta-related cytokine, have been reported as a biomarker in cardiovascular disease as well as a strong prognosticator of heart failure (Kempf et al. 2007; Kempf and Wollert 2009). We also found GDF15 highly upregulated in hiPSC-CMs after ETP treatment. Altogether, these findings suggest that the gene expression profiles observed using our in vitro system are very similar to those observed in patients with heart conditions and therefore offer human-relevant model which can be used as an effective alternative for costly and time consuming animal studies.

In addition we also identified 58 deregulated genes consisting 33 upregulated and 25 downregulated genes in hPSC-CMs after ETP treatment. GO and KEGG pathway analysis confirmed most upregulated genes are enriched in GO categories like positive regulation of apoptotic process, regulation of cell death, and mitochondria organization, whereas most downregulated genes were enriched in GO categories like cytoskeletal organization, muscle contraction and Ca^2+^ ion homeostasis. Moreover we also found upregulation in 5 miRNAs (has-miR-486-3p, has-miR-34c-5p, has-miR-4423-3p, has-miR-182-5p, and has-miR-139-5p) which play role in muscle contraction, Arginine and proline metabolism and Hypertrophic cardiomyopathy (HCM). Immunostaining and Transmission Electron Microscopy also confirmed the cytoskeletal and mitochondrial damage in hPSC-CMs treated with ETP. Using xCELLigence Real-Time Cell Analyser (RTCA), we show that apoptosis inhibitor, Pifithrin-α, protect hPSC-CMs from ETP-induced cardiotoxicity, whereas hPSC-CMs treated with ferroptosis inhibitor, Liproxstatin-1, showed significant recovery during wash out period. Altogether, these observations show that ETP on one hand induces cytoskeletal deterioration in hPSC-CMs leading to cardiomyopathy, while on the other hand upregulates apoptosis and programmed cell death signaling as a result of DNA and mitochondrial damage.

The hPSC-CMs are specialized muscle cells which possess highly organized structures of myofilaments made up of repeating micrometer-sized units, termed sarcomeres; which are involved in mechanical cardiac contraction. Sarcomeric proteins of thin filament, thick filament and Z-disc are important in formation of basic contractile units and any alterations in their functions can result in cardiomyopathy or heart failure (Hwang and Sykes 2015). Our qRT-PCR data showed significant downregulation in mRNA levels of sarcomere genes including TCAP, MYL2, MYL3, MYH7, ACTA1, and LDB3 in hPSC-CMs treated with ETP. Immunostaining for sarcomeric proteins and transmission electron microscopy further confirmed the deterioration of myofilaments in hPSC-CMs treated with ETP compared to that of untreated CMs. High dose of ETP in particularly showed severely disorganized myofilaments.

Although regulation of Ca^2+^ ion homeostasis is one of the essential functional elements during cardiac contraction and is tightly regulated by Ca^2+^ ion channels and Ca^2+^ binding proteins, cytosolic Ca^2+^ overload leads to ischemic myocardial injury and death (Marsh and Smith 1991). The ryanodine receptors (RyRs) are intracellular calcium-release channels, expressed in cardiomyocytes and downregulation of RyRs has been reported to contribute to impaired contractility (Go et al. 1995). Our data showed clear downregulation in mRNA levels of important Ca^2+^ regulating genes including RyR2, CASQ2, ATP1A2 and DMD. The hiPSC-CMs treated with ETP at 10 µM and 15 µM showed higher Ca^2+^ transient amplitude, a slowing in reaching the [Ca^2+^] peak as well a prolonged presence of Ca^2+^ in the sarcoplasm. In contrast, 30 µM ETP caused an acceleration of the ascending phase of the transient. These findings further confirm that ETP has arrhythmogenic and cardiotoxic potential.

Like other DNA damaging topoisomerase inhibitors, ETP induces apoptosis in a variety of cell types and p53 has been shown to play important role in ETP-induced apoptosis (Grandela et al. 2008). Induction of apoptosis in hPSC-CMs is known to be involved in establishment of cardiotoxicity (Arola et al. 2000; Kang 2001), whereas inhibitors of apoptosis like Pifithrin-α have been shown to protect cardiomyocytes from DOX-induced apoptosis and acute cardiotoxicity (Liu et al. 2004) Our data show that ETP induces upregulation of genes involved in DNA damage response and apoptosis like BAX, FAS, GADD45A, and RGCC. It also leads to overall increase in ROS production in hPSC-CMs suggesting activation of apoptosis signaling. However, when hPSC-CMs were treated simultaneously with ETP and Pifithrin-α, we observed no significant improvements in hPSC-CMs viability indicating apoptosis might not be the only pathway involved in ETP-induced cardiotoxicity.

Mitochondria occupy 30% of cell volume in adult cardiomyocytes and plays essential role in cardiac muscle contraction and balance of Ca^2+^ and K^+^ concentrations. Mitochondrial biogenesis involving balanced mitochondrial fission and fusion plays an important part in cardiomyocytes contractility, whereas increased mitochondrial fission leads to mitophagy as a result of increase in cytochrome c leakage (Givvimani et al. 2015; Pennanen et al. 2014). Damage to mitochondria has been shown to result in disruption of Ca^2+^ homeostasis and oxidative phosphorylation, increased production of reactive oxygen species (ROS), reduction in ATP production and activation of apoptosis signaling (Figueira et al. 2013; Piquereau et al. 2013; Shen and Jennings 1972). In fibroblasts, ETP induces apoptosis and cell death via mitochondrial-dependent activation of p53 (Jamil et al. 2015; Karpinich et al. 2002). In agreement with these observations, our data also showed increased mitochondrial fission and upregulation in SPATA18, NQO1, GLS2 and GPX1 after ETP treatment. Staining of hPSC-CMs with DHE further confirmed increased ROS production in ETP-exposed hiPSC-CMs. Using JC-1 staining, we also show that ETP treatment causes alteration in mitochondrial membrane potential (*m*∆*ψ*) indicating increased number of apoptotic hPSC-CMs and reduced ATP production. Collectively, these alterations in mitochondrial structural and functional properties indicate possible activation of ferroptosis pathway in ETP-treated CMs. Our RTCA data show that treatment of Liproxstatin-1 helps hPSC-CMs to recover from ETP-induced increase in beating rate and beating amplitude during drug washout. Further studies could shed more light on the association of ferroptosis and cardiotoxicity induced by anti-cancer drugs.

In summary, cardiovascular complications pose a rare but potential fatal adverse effect of ETP chemotherapy and should be carefully addressed, especially in patients with additional cardiovascular risk factors or when used in combination therapy. Our findings suggest that damages to mitochondria are a major contributing factor involved in ETP-induced cardiotoxicity. Even though, the exact mechanism by which ETP affects mitochondrial integrity is not fully understood, we hypothesize that damages to mitochondria and impaired mitochondrial bioenergetics and calcium handling are the major factors involved in ETP-induced cardiotoxicity. Our data also indicate that cardiotoxic effects of ETP could be synergistically increased in presence of other DNA damaging or cytotoxic agent and should be taken under consideration before starting the drug administration. In addition, in the present study, we have established an in vitro cardiotoxicity methodology for the monitoring of early as well as chronic cardiotoxicity events at the cellular, functional and genomic level.

## Electronic supplementary material

Below is the link to the electronic supplementary material.

Supplementary material 1 (XLS 471 KB)

Supplementary material 2 (PDF 2103 KB)
