# Supplementary material for: Cell death mechanisms of the anti-cancer drug etoposide on human cardiomyocytes isolated from pluripotent stem cells
Source: Arch Toxicol. 2018 Feb 3;92(4):1507–24. doi: 10.1007/s00204-018-2170-7 (PMC5882643; doi:10.1007/s00204-018-2170-7)
Supplement: Supplementary file 2 — Supplementary material 2 (PDF 2103 KB) [file 204_2018_2170_MOESM2_ESM.pdf]

Figure S1

S1A

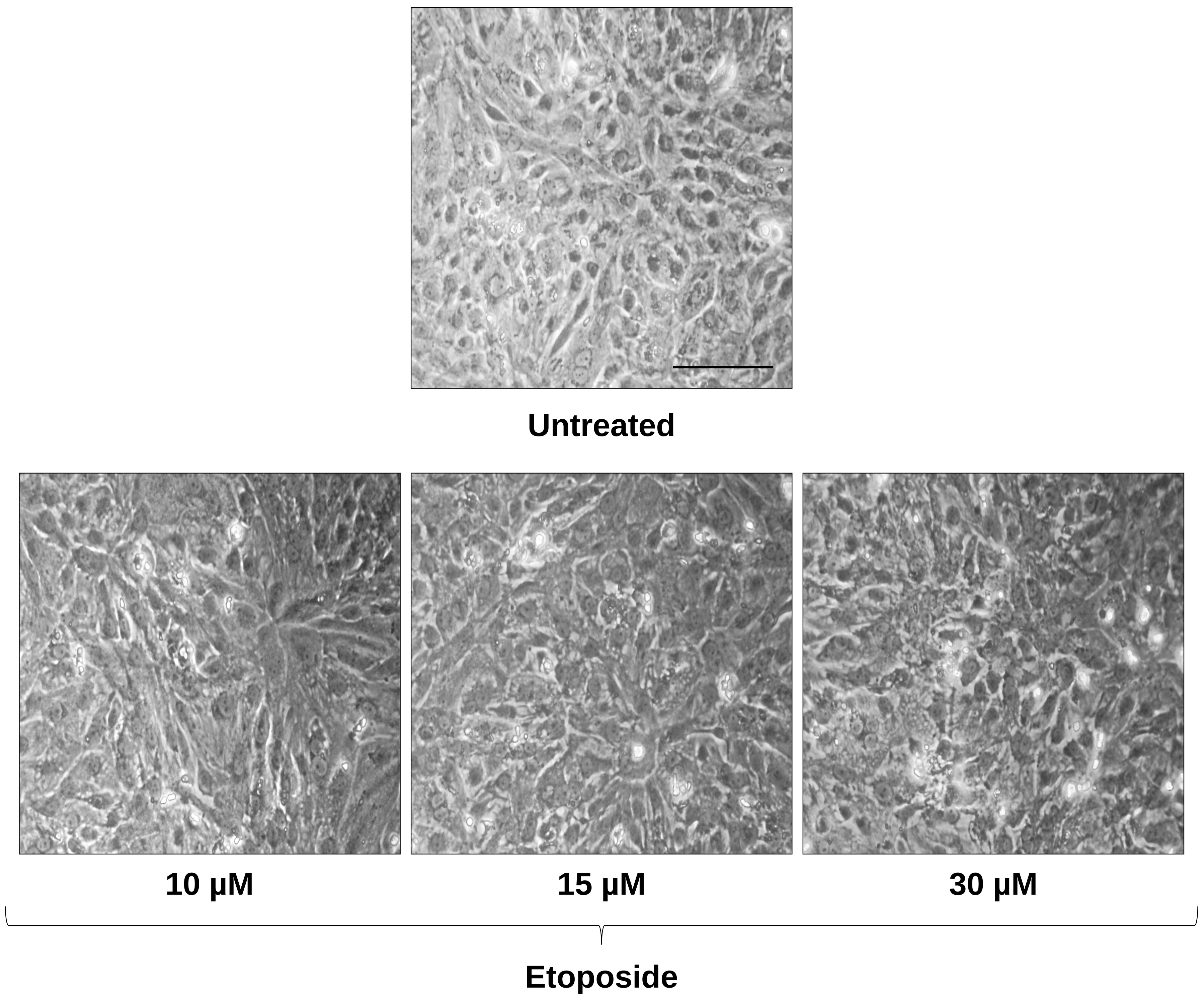

S1B

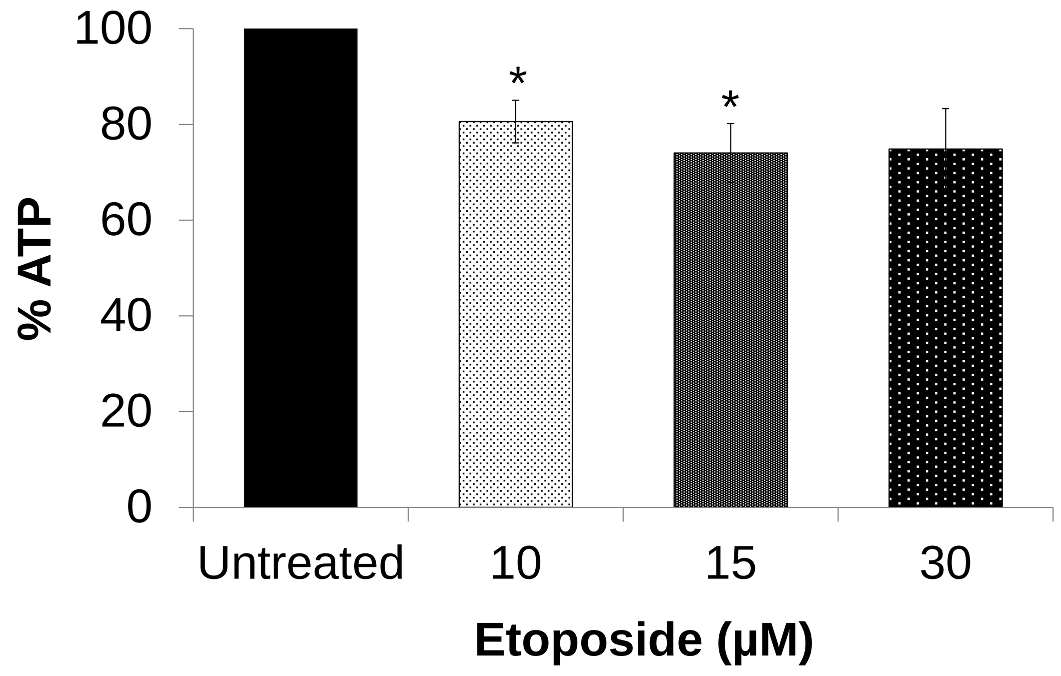

Supplementary figure 1. A) Influence of single high-dose of ETP (10 µM, 15 µM and 30 µM) on cell density and cell morphology. The scale bar represents 50 µm. B) Influence of single high-dose of ETP (10 µM, 15 µM and 30 µM) on intracellular ATP levels in hiPSC-CMs. (n = 3, error bars represent ± SEM) (t test, *$p \leq 0.05$).

Figure S2

S2A

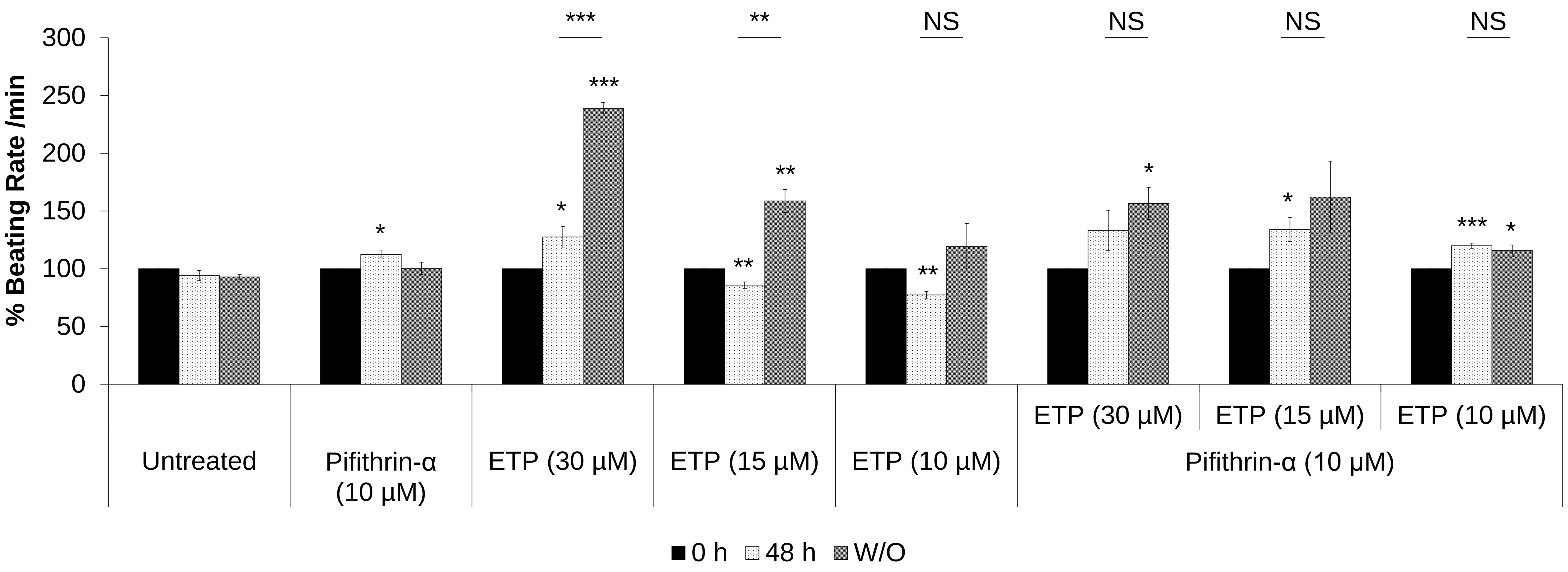

S2B

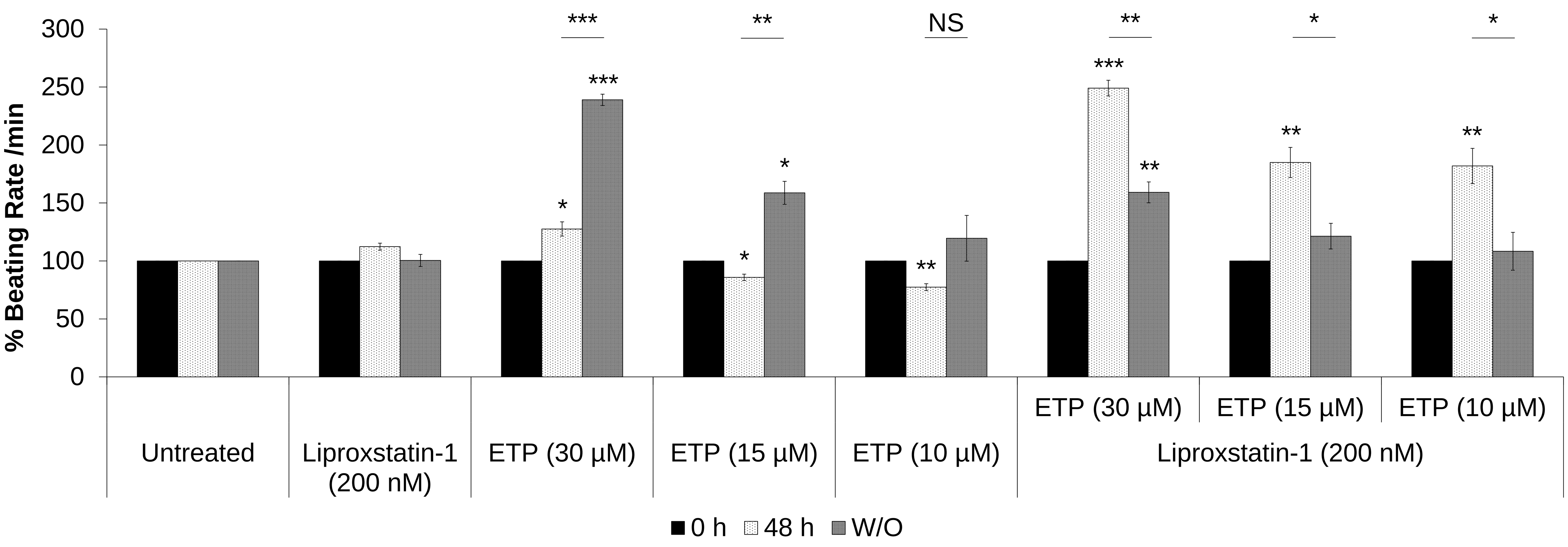

Supplementary figure 2. A) The hiPSC-CMs were co-treated with ETP and 10 µM Pifithrin-α (apoptosis inhibitor) for 48 h. Real time data of CMs beating rate was obtained using xCELLigence RTCA system. Representative graphs display % beating rate values. (n = 3, error bars represent ± SEM) (t test, *p ≤ 0.05, **p ≤ 0.01, ***p ≤ 0.001, NS, non-significant). B) The hiPSC-CMs were co-treated with ETP and 200 nM Liproxstatin-1 (ferroptosis inhibitor) for 48 h. Real time data of CMs beating rate was obtained using xCELLigence RTCA system. Representative graphs display % beating rate values. (n = 3, error bars represent ± SEM) (t test, *p ≤ 0.05, **p ≤ 0.01, ***p ≤ 0.001, NS, non-significant).

Supplementary table 1. The fold change values for 84 genomic markers after Etoposide treatment

	Etoposide (µM)		
	10	15	30
TNNI3	0.3	-1.1	-1.7
MYL2	0.3	-1.3	-3.2
MYL3	-1.6	-2.0	-5.1
ACTA1	-1.7	-2.0	-3.3
TNNT1	1.7	1.6	1.6
DES	-1.5	-1.7	-3.2
TCAP	1.3	-0.3	-2.1
PGAM2	-0.4	-0.5	-1.5
HRC	-0.5	-0.5	-1.7
MYOM2	-1.4	-1.5	-2.1
MB	-0.3	-1.1	-1.4
FHL2	-1.4	-1.7	-3.7
MYH6	0.3	1.1	1.2
MYH7	-1.6	-1.6	-3.8
GJA5	-1.9	-2.0	-2.3
ANK2	-1.3	-1.1	-1.2
ARG2	-2.3	-2.3	-2.3
EDNRA	-1.2	-1.2	-1.6
LDB3	-0.5	-1.2	-1.8
CASQ2	-0.6	-1.5	-3.2
DMD	-1.5	-1.6	-2.1
NRAP	1.3	1.3	-1.9
UCP2	-2.3	-2.3	-2.9
COX6A2	-1.3	-1.6	-3.6
ATP1B4	1.8	1.9	0.7
ATP1A2	-1.2	-1.3	-2.1
KCNQ1	-1.2	-1.2	-1.5
KCNK3	0.6	1.4	1.4
KCNN2	-1.4	-1.7	-2.7
KCNH2	0.5	1.3	1.3
SCN2B	0.6	1.3	1.1
SCN5A	0.4	1.1	-1.0
SLC8A1	-1.5	-1.6	-2.6
CACNA2D2	-1.5	-1.3	-1.3
PYGM	-2.6	-2.9	-3.5
CKM	-1.9	-2.6	-5.0
KRT19	1.4	1.6	2.0
ANK1	7.9	10.0	10.4
PDLIM3	2.9	3.0	2.9
ABRA	1.9	2.8	3.0
MYOT	2.4	2.5	2.9
SCN4B	3.1	3.1	2.9
TRPV1	0.7	1.7	2.5
GLS2	2.6	3.3	4.8
PDK1	-1.8	-2.0	-2.2
PDK4	0.6	1.3	2.1
BAX	3.1	3.5	4.0
CASP3	1.3	1.5	1.6
MURC	-0.3	-0.4	-1.4
POSTN	2.1	1.9	1.6
ERBB3	-1.5	-1.9	-2.3
JAK2	1.1	-1.2	-0.7
IGFBP3	1.3	1.3	1.4
PPP1R3A	-0.4	-0.4	-0.5
RYR2	-1.7	-1.7	-2.5
IRX4	-1.4	-1.4	-1.8
CORIN	-1.5	-1.6	-1.7
CACNA1G	-1.5	-1.8	-1.7
ASPH	-0.4	-0.3	-1.1
STC1	0.0	0.6	2.8
PRDM16	-0.7	-0.6	-1.4
CALM1	1.4	1.5	1.8
ZMAT3	2.5	2.8	3.1
GDF15	53.1	72.1	89.3
ACE2	2.0	2.1	1.9
AVPR1A	6.8	10.0	11.8
FAS	9.1	10.0	14.8
PRKX	4.2	5.1	5.9
DUSP4	4.3	5.8	10.2
GPNMB	0.9	1.8	1.8
SPATA18	4.8	5.4	6.1
THBS4	1.9	1.9	1.8
RGCC	2.1	2.7	4.7
NRG1	2.8	3.8	4.3
CTGF	3.3	4.1	5.9
KCNJ2	1.4	1.5	1.4
F11R	2.1	2.5	3.1
TPM1	-1.0	-1.2	-1.7
KLF5	0.8	1.8	1.9
GADD45A	3.4	3.9	4.2
GPX1	2.0	2.4	2.8
LIF	7.2	9.7	14.7
NQO1	1.1	1.3	1.9
OXR1	1.1	1.2	1.2

Supplementary table 2. The fold change values of 14 miRNAs after Etoposide treatment

	Etoposide (µM)		
	10	15	30
hsa-miR-486-3p	5.1	5.2	6.4
hsa-miR-34c-5p	2.2	2.2	2.1
hsa-miR-4423-3p	2.3	2.2	2.0
hsa-miR-182-5p	1.4	1.7	2.9
hsa-miR-139-5p	1.2	1.6	2.3
hsa-miR-34a-3p	1.8	1.6	1.4
hsa-miR-1303	1.2	1.6	1.7
hsa-miR-187-3p	1.3	1.1	1.0
hsa-miR-486-5p	1.3	1.0	1.0
hsa-miR-34c-3p	1.0	1.2	1.1
hsa-miR-212-3p	0.8	0.8	0.7
hsa-miR-4298	0.8	0.9	0.8
hsa-miR-3911	0.9	0.9	0.8
hsa-miR-675-5p	0.7	0.7	0.7
